# The patients’ perspective: living with lupus in Austria

**DOI:** 10.1007/s00508-017-1175-1

**Published:** 2017-02-22

**Authors:** Georg Stummvoll, Tanja Stamm

**Affiliations:** 1 Department of Rheumatology, Medical University of Vienna, Spitalgasse 23, 1090 Vienna, Austria; 2 Center for Medical Statistics, Informatics and Intelligent Systems, Medical University of Vienna, Vienna, Austria

**Keywords:** Systemic lupus erythematosus (SLE), Patients’ perspective, Diagnosis

## Abstract

Systemic lupus erythematosus (SLE) is a heterogeneous disease with a vast variety of clinical manifestations. Timely diagnosis is important for gaining access to specific therapy and care. In this survey, we asked SLE patients with an established diagnosis to report about their personal history and their daily life with SLE in order to gain knowledge about diagnostics, treatment pathways and potential problems in daily living and functioning. In most cases, the diagnosis of SLE was made by a specialist in rheumatology or dermatology. Of the patients 41.5% were diagnosed within the first year after onset of disease symptoms, while 37.3% of the patients waited for 3 or more years for the final diagnosis of SLE. Interestingly, we found no differences with respect to patients living in urban or rural areas. Specific therapy worked well in many but not in all patients: the majority of patients reported problems with paid work, social life and leisure activities including traveling. Patients reported a need for better information for the general public about SLE. In addition, they wanted be better informed themselves*.* Despite all successful efforts in recent years, there is still room for improvement with respect to early diagnosis, early start of specific therapy and for better information of the public on the mysterious disorder named SLE.

Systemic lupus erythematosus (SLE) is a multiorgan disorder and can present with different clinical features due to a variable involvement of internal organs, the musculoskeletal system, the nervous system, the skin or the hematopoietic system. One of the main difficulties in SLE is to make a correct and timely diagnosis since diagnosis criteria are still missing and the existing classification criteria are not perfectly suitable for (early) diagnosis, despite some changes in the recent past [1–3]. In addition, many common SLE symptoms are relatively unspecific (such as fever and anemia) and SLE patients often suffer from combinations of various symptoms or organ manifestations.

The prevalence of SLE in European studies lies between 25 and 91 per 100,000 persons which would account for an estimated approximation of 4000 SLE patients in Austria with 90% of being female [4]. As in other countries, Austrian SLE patients are treated by different specialists because of historical reasons and/or because of the leading set of symptoms. Recently, a Swiss group reported a cross-sectional analysis of clinical characteristics and treatments across different medical disciplines in Switzerland [5]. Due to the heterogeneity of the disease and the possibility of life-threatening organ manifestations, SLE patients are preferably treated in larger (tertiary) centers which offer a multidisciplinary approach, but especially milder forms are also treated in an extramural setting. Given the fact that early diagnosis is important but difficult, we asked our patients how long they waited for a final diagnosis, which doctor actually made it, how many physicians/specialists they had to visit before a final diagnosis was made and if there were differences between patients living in rural or urban areas. Although SLE can affect essentially all organ systems, up to 95% of SLE patients suffer from inflammation, pain and malfunction of the musculoskeletal system leading to activity limitations and participation restrictions in daily life [6, 7]. In a previous qualitative study, we explored the array of concepts important to patients with chronic SLE and compared these to instruments assessing disease activity, damage and health status. A wide range of concepts were identified; however, only a small number were found to be covered by the instruments commonly used in clinical practice and research [6]. Therefore, the patients’ perspective on rheumatic diseases came into the focus of interest, but data in general and such on Austrian patients, in particular, are scarce. In this survey, we asked SLE patients with an established diagnosis to report about their personal history and their daily life with SLE in order to gain knowledge about diagnostics, treatment pathways and potential problems in daily living and functioning.

## Methods

### Questionnaire

A survey was conducted with a self-developed questionnaire that we piloted and adapted after a preliminary test with 10 patients.

### Patients

Patients were informed about this survey via flyers and posters which were on display in outpatient wards and doctors’ offices of dermatologists and rheumatologists. In addition, we created the homepage www.lebenmitlupus.org (i. e. living with lupus) as a tool of information for patients and their relatives. In order to participate in our survey, questionnaires could be submitted either online or per mail. In order to ensure anonymity and to keep the questionnaire simple, we provided ticking options for most of the answers, asking either for a yes/no decision or asked to choose from four different grades of approval. We chose this approach since anonymity was of high importance for questions related to employment (e. g. does your employer know about your disease?) or social situation and not easily achieved in this rare disease. Recruitment lasted from 1 January to 31 December 2013. The questionnaires were distributed and processed by Public Health PR, Vienna (http://www.publichealth.at/), and the project “Leben mit Lupus” (i. e. living with lupus) was financially supported by GlaxoSmithKline. This questionnaire survey was done in agreement with local laws (Wiener Krankenanstaltengesetz §15 Abs. 3, as confirmed by the ethics committee of the City of Vienna, 16-261-VK, 2016).

### Statistical analysis

The questionnaire consisted of 30 questions in 5 categories (i.e. route to diagnosis, therapy, social environment, information and communication and demographic data). Only fully completed questionnaires were used for the analysis. We used the following procedure to check that patients with SLE did not fill in the questionnaire more than once: questionnaires with identical answers submitted within 1 h were counted only once. Descriptive statistics were used to describe the answers of the patients. For categorical variables (e. g. sex/gender, medication), the absolute frequency (*n* =) and the percentage frequency (%) were calculated and reported.

## Results

A total of 125 questionnaires were received and of these, 118 were complete and could be included in the analysis. The demographic data of the participants are reported in Table 1.

**Table 1:** Patients’ characteristics, first symptoms, diagnosis modalities and medication. All information derived from anonymized questionnaires

Questionnaires received		118		
**Patients**				
Sex	Female/male	91/9%		
Age (years)	0-15 years	0%		
	16-25 years	17%		
	26-35 years	22%		
	36-49 years	43%		
	>50 years	18%		
Domicile	Urban/rural	62/48%		
Education	Compulsory	59%		
	High school	20%		
	University	21%		
**Medication**	NSARD	22.0%		
	Steroids	62.7%		
	Antimalarial drugs	55.1%		
	Immunomodulators	44.1%		
	Biologicals	7.6%		
**First symptoms**	**Fatigue**	**61.9%**	Headache	22.0%
	**Arthralgia/arthritis**	**60.2%**	Anemia	20.3%
	**Fever**	**41.5%**	Swollen LN	16.1%
	**Rash**	**37.3%**	Angina pectoris	15.3%
	UV intolerance	28.0%	Thrombosis	13.6%
	Depression	25.4%	Dyspnea	12.7%
	Raynaud’s phenomenon	23.7%	Oral/genital ulcers	10.2%
	Edema	23.7%		
**Final diagnosis by**			**Domicile**	
			***Urban/rural***	
	Rheumatologist	50.9%	54/51%	
	Dermatologist	22.0%	25/19%	
	Internist	13.6%	8/23%	
	General practitioner	6.8%	7/7%	
	Neurologist	3.4%	6/0%	
**Doctors involved (** ***n*** **)**	1-3	43.2%	42/44%	
	4-6	36.4%	37/38%	
	7-9	6.8%	8/4%	
	>10	13.6%	14/13%	
**Time to diagnosis**		**All patients**	**Domicile**	**Education**
			***Urban/rural***	***University/compulsory***
	<1 year	41.5%	42/40%	44/35%
	1-3 years	21.2%	22/20%	26/25%
	3-6 years	9.3%	5/16%	9/5%
	6-12 years	16.1%	16/16%	15/25%
	>12 years	11.9%	14/9%	6/10%

*NSARD* nonsteroidal antirheumatic drugs, *LN* lymph nodes, *UV* ultraviolet

Most patients presented with fatigue, arthralgia, rash and fever of unknown origin and thus with typical but relatively unspecific symptoms [8]. An early diagnosis (within the first year) was made in 41.5%, an additional 21.2% had SLE diagnosis within the first 3 years, but for more than one third of SLE patients, diagnosis took more than 3, in some cases even more than 12 years (Table 1). Unexpectedly, early diagnosis was not higher in patients living in urban than in those living in rural areas (42% vs. 40%, respectively), but appeared slightly higher in patients with a higher education: 70% of patients in the best educated group had SLE diagnosis within 3 years compared to 60% in the lowest educated group (Table 1, Fisher’s exact test *p* = *n*. s.) and also the percentage of patients with delayed diagnosis (>6 years) was slightly higher in less educated patients (35% vs. 21% in the best educated group).

**Fig. 1:**
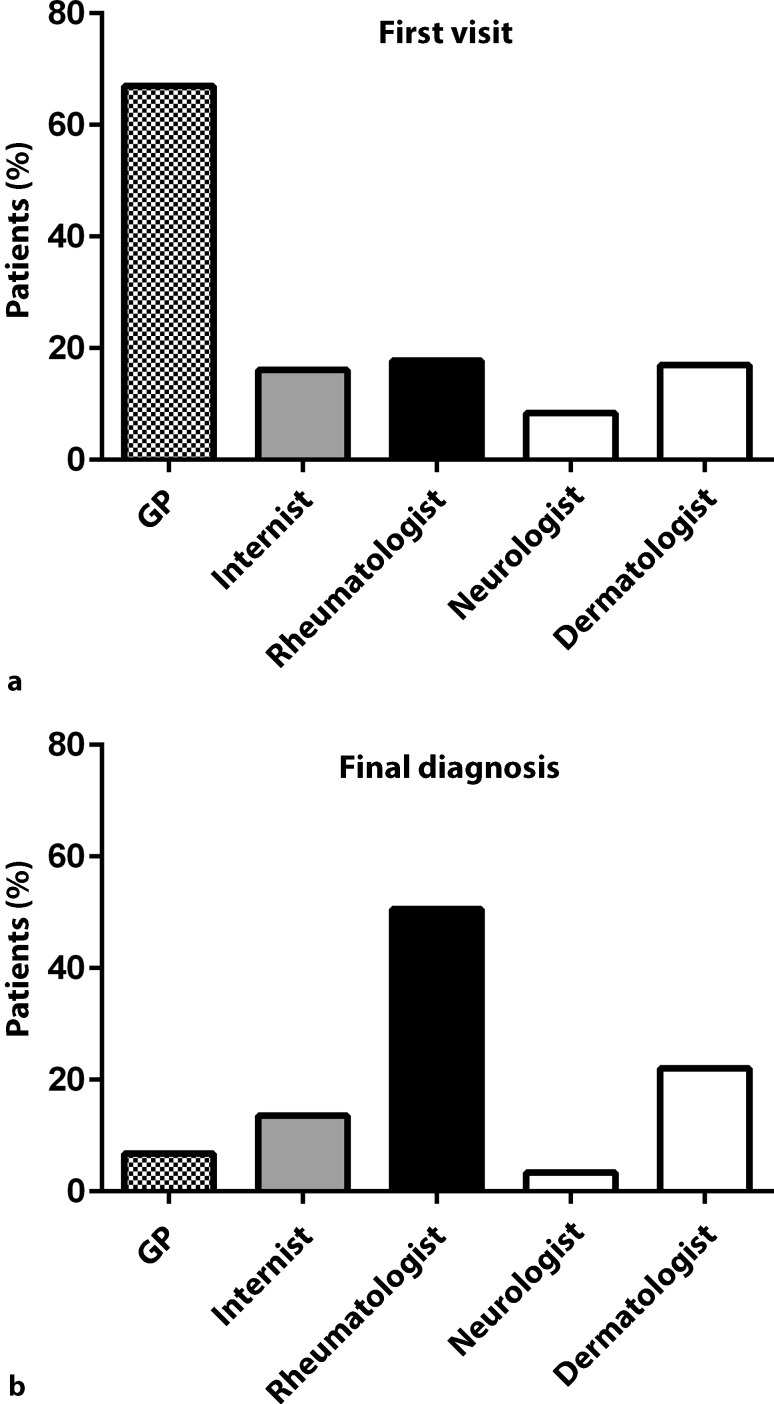
First visit and final SLE diagnosis. 67% of patients had their first visit for SLE symptoms at their general practitioner (GP, **a**), but diagnosis was made by the respective specialists, mainly by rheumatologists, dermatologists and general internists (**b**)

In general, most patients were seen first by a general practitioner (67%), but the final diagnosis was made by a rheumatologist (50.9%) or dermatologist (22.0%) (Fig. 1; Table 1). In 43% of patients 1–3 doctors were involved, the rest needed more contacts for SLE diagnosis. Again, there was no difference between patients from urban or rural areas (Table 1).

**Fig. 2:**
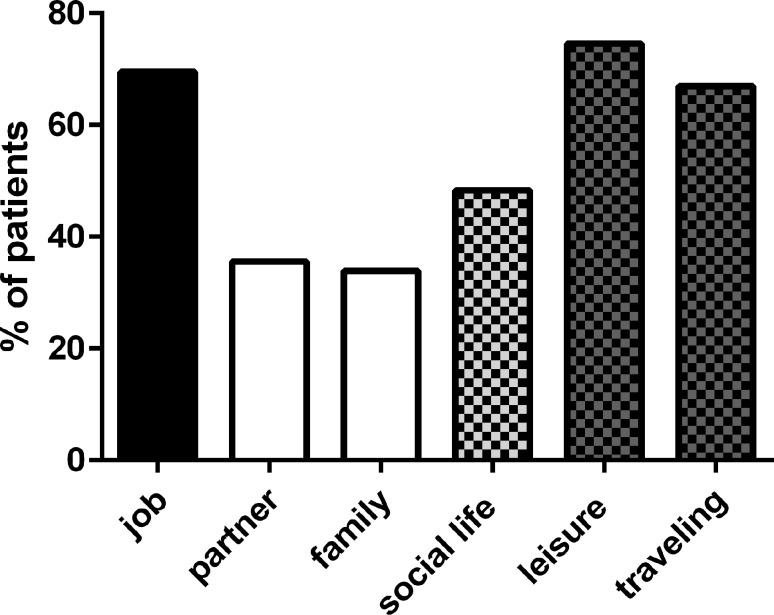
Social life. Patients were asked for disease-related disadvantages with respect to their job, social situation in general, special situation with partner or family, traveling and leisure time activities. Values are given as the percentage of positive reports

Patients received standard of care therapy (SOC), including non-steroidal antirheumatic drugs (NSARD), steroids, antimalarial drugs and other immunomodulators and, in a small number, also biologicals (Table 1). Of the patients 66% reported an improvement of their physical symptoms under therapy. Consequently, the number of patients rating their overall physical condition “very poor” was reduced from 55% to 18% under therapy, yet only 17% reached a state where they felt “good” (rating possibilities were good, moderate, poor and very poor). In addition, 54% of patients also reported an improvement in their mental condition. In summary, SOC worked, but did not lead to remission in all patients. As expected from personal contact and from the literature [9, 10], the majority of patients reported problems with paid work, social life and leisure activities including traveling (Fig. 2). Interestingly, most of the same patients did not report difficulties in their partnership or family. More than 80% lived in a partnership/marriage. It is noteworthy that more than 69% reported problems with respect to their job and social situation at their working place, but only 57% informed their employer about their disease and 67% informed at least their closest colleagues. Comparing patients with the highest and the lowest level of education, both groups reported very similar frequent problems (74% and 77%, respectively). Nevertheless, the vast majority of SLE patients explained the wish that the rest of the population should be better informed about the disease per se (82.2%).

Patients received information about SLE primarily from their doctors (82.2%) and from web-based sources (68.6%), also from brochures (23.7%), other print media (14.4%), or from self-help groups (16.1%), but rarely from books (5.9%) or at pharmacies (1.7%).

## Discussion

This survey describes the current situation of SLE patients in Austria related to the course of disease and the problems in daily life. The most unexpected findings were the high proportion of patients having a latency period of more than 3 years to being diagnosed with SLE, that living in urban or rural areas did not make a big difference with this respect, and that only a small number of patients rated their condition as “good” despite intensive therapy.

Our study has some limitations based on the nature of the questionnaire, which we designed in the attempt to keep it short, simple and anonymized. On the other hand, patients trusted our efforts and accepted the questionnaire despite its limitations. The sample size is to our knowledge the largest so far dealing with the perspective of Austrian SLE patients on their personal history with SLE.

Although patients presented with typical (although relatively non-specific) symptoms, more than one third of SLE patients waited 3 years or longer for a final diagnosis and, thus, specific therapy. As in other rheumatic diseases, early diagnosis and consequently early therapy in SLE is necessary and should be improved in order to avoid damage brought about by the disease itself or by disease-related comorbidities [11]. As in most cases, SLE diagnosis has been made by a specialist (rheumatologist, followed by dermatologist and unspecified specialist in internal medicine), an easy access to specialized persons is essential, as it is with other inflammatory rheumatic diseases [12, 13]. Recently, an Austrian group analyzed the interface management between general practitioners and rheumatologists in a survey and tried to define a concept for future joint recommendations [14]. Interestingly and against our expectations, there were no differences in time to diagnosis between patients form urban and rural areas. An explanation might be that general practitioners from rural areas (who are the first to see SLE patients in most cases) refer their patients to specialists in internal medicine, rheumatology or dermatology (who make the final diagnosis) in a very similar manner as their colleagues do in urban environments.

Although SLE therapy has gained a lot of options in recent years, there is still a much too high number of patients regarding their physical status as “poor” or “very poor” despite therapy. In addition, patients also still experience disadvantages in crucial aspects of their daily life, including their job and general social situation at their working place as well as during traveling and leisure activities. These problems appear to be aggravated in situations when patients have to cooperate with persons who are not informed about their disease or about SLE in general, since many patients do not inform their employers or closer social environment. Consequently, these patients see a need for better information of the general public about SLE and they also want to be better informed themselves.

Many efforts have been made in the last years to make SLE therapy more effective and more precise as well as to understand the disease from the patients’ perspective and to design better tools for assessing SLE. Despite all successful efforts in recent years, there is still room for improvement with respect to early diagnosis, early start of specific therapy and for information of the public on the mysterious disorder named SLE.
